# Non Stationary Multi-Armed Bandit: Empirical Evaluation of a New Concept Drift-Aware Algorithm

**DOI:** 10.3390/e23030380

**Published:** 2021-03-23

**Authors:** Emanuele Cavenaghi, Gabriele Sottocornola, Fabio Stella, Markus Zanker

**Affiliations:** 1 Dipartimento di Informatica, Sistemistica e Comunicazione, University of Milano-Bicocca, 20126 Milano, Italy; e.cavenaghi@campus.unimib.it (E.C.); stella@disco.unimib.it (F.S.); 2 Facoltà di Scienze e Tecnologie Informatiche, Free University of Bozen-Bolzano, 39100 Bolzano, Italy; Markus.Zanker@unibz.it

**Keywords:** machine learning, time-series analysis, multi-armed bandit, Thompson Sampling, non-stationary multi-armed bandit, concept drift

## Abstract

The Multi-Armed Bandit (MAB) problem has been extensively studied in order to address real-world challenges related to sequential decision making. In this setting, an agent selects the best action to be performed at time-step *t*, based on the past rewards received by the environment. This formulation implicitly assumes that the expected payoff for each action is kept stationary by the environment through time. Nevertheless, in many real-world applications this assumption does not hold and the agent has to face a non-stationary environment, that is, with a changing reward distribution. Thus, we present a new MAB algorithm, named *f-Discounted-Sliding-Window Thompson Sampling* (*f-dsw TS*), for non-stationary environments, that is, when the data streaming is affected by *concept drift*. The *f-dsw TS* algorithm is based on Thompson Sampling (TS) and exploits a discount factor on the reward history and an arm-related sliding window to contrast concept drift in non-stationary environments. We investigate how to combine these two sources of information, namely the discount factor and the sliding window, by means of an aggregation function f(.). In particular, we proposed a pessimistic (f=min), an optimistic (f=max), as well as an averaged (f=mean) version of the *f-dsw TS* algorithm. A rich set of numerical experiments is performed to evaluate the *f-dsw TS* algorithm compared to both stationary and non-stationary state-of-the-art TS baselines. We exploited synthetic environments (both randomly-generated and controlled) to test the MAB algorithms under different types of drift, that is, sudden/abrupt, incremental, gradual and increasing/decreasing drift. Furthermore, we adapt four real-world active learning tasks to our framework—a prediction task on crimes in the city of Baltimore, a classification task on insects species, a recommendation task on local web-news, and a time-series analysis on microbial organisms in the tropical air ecosystem. The *f-dsw TS* approach emerges as the best performing MAB algorithm. At least one of the versions of *f-dsw TS* performs better than the baselines in synthetic environments, proving the robustness of *f-dsw TS* under different concept drift types. Moreover, the pessimistic version (f=min) results as the most effective in all real-world tasks.

## 1. Introduction

In the context of sequential decision making, the Multi-Armed Bandit (MAB) problem has been extensively studied by researchers in the field of reinforcement learning, since it was firstly introduced in the middle of last century by Robbins [1]. The MAB problem [2], is used to represent the exploration-exploitation dilemma in sequential decision problems, that is, how to acquire knowledge about the set of available actions while exploiting the most profitable ones. In particular, during each round of the sequential decision problem, an agent selects one among the *K* available actions (called arms), and receives a reward (or payoff) proportional to the goodness of its choice. The goal of the agent is to maximize the collected reward (or minimizing the regret) over the complete set of *T* rounds. In the literature, different policies have been studied to solve this problem, with Thompson Sampling (TS), being one of the most used and successful. Through the years, there have been many practical applications of MAB in real-world scenarios, that justify the wide interest in the framework. For instance, in the field of clinical trials [3,4], online advertising [5,6], dynamic routing [7], A/B testing [8], and recommendation systems [9,10].

Generally, MAB techniques explore the environment during the early stages of the sequential decision problem to find the best arm to play. After this exploration phase, sufficient knowledge over the different arms is acquired and the algorithm can exploit the best arm to maximize the collected rewards (or to minimize the regret). Thus, standard MAB formalization implicitly assumes that the reward distributions of different arms are stationary (i.e., do not change over time). However, in real life, it is common to find applications where this assumption does not hold and the reward distributions evolve over time. A typical example is the one of online recommendations, in which products/contents are subjected to trends or seasonality [11]. In this setting, a “stationary” policy may fail to promptly detect the changes happening on the reward distributions through time. This leads to sub-optimal decisions by the MAB agent (e.g., exploiting an arm, whose payoff has decreased over time). Consequently, an improved policy is needed that takes the potential shifts in reward distributions into account and adapts the agent’s choices accordingly. This formulation of the MAB problem, in which reward distributions may change over time, is said to be *Non-Stationary* [12] and it is still under-explored. In time series analysis, this type of non-stationary behavior over the action (class) distributions is referred to as *Concept Drift* [13]. In real-world scenarios, concept drifts may happen at any point in time due to different reasons such as a switch in the costumers’ purchasing behavior for winter clothes due to the approaching spring. Thus, the modification of the underlying reward distributions of the available actions (e.g., recommending for winter gloves), requires the MAB algorithm to quickly adapt to the evolving situation. Concept drifts could be of various nature, namely *abrupt* (or *sudden*), *incremental*, *gradual* as well as *reoccurring* [14]. We borrow the formal definitions of these drift types from time series analysis literature and adapt them to the context of non-stationary MAB.

In this paper we propose *f-Discounted-Sliding-Window Thompson Sampling (f-dsw TS)*), a novel MAB algorithm for solving sequential decision making problems in non-stationary setting (i.e., under concept drift). Specifically, the method extends TS policy with both a *discount factor* over the estimated reward distributions, and a *sliding window* to remember the outcome of the last *n* actions taken by each arm. The discount is applied to increase the uncertainty about the past knowledge of the actions’ payoffs, by increasing the variance associated to each arm. In this way, we force the agent to maintain a certain degree of exploration given the higher variance over the historic reward estimations, than in the standard version of Thompson Sampling. The sliding window ensures that the agent can adapt faster to the potential concept drift, because it takes into account only the last *n* rewards for estimating the payoff of each arm. Unlike [15], that exploits a single window for all the actions available to the agent, regardless of the arm that was played, we made the decision to use one window for each arm. Thus, we keep *K* different lists with the last *n* actions played, on which to update the estimations of the agent over the different reward distributions.

We prove the effectiveness of *f-dsw TS* with a rich set of numerical experiments. In this empirical evaluation, we compare the performances of *f-dsw TS* to other TS competitors, both in synthetic and real-world environments. The synthetic data serve to evaluate and compare the algorithms under different controlled settings, as well as in totally randomized ones. Specifically, we design three custom environments: an environment in which the expected payoff for the optimal arm suddenly decreases, an environment in which the payoff for a sub-optimal arm suddenly increases (becoming the new optimal), and a stationary environment (i.e., in which the reward distributions are kept constant for all the rounds). Along with these, we define experiments on totally randomized environments, with random initial reward distributions and random changes, for the two main drift types (i.e., sudden drift and incremental drift). For the real-world evaluation, we consider an heterogeneous set of four case studies, derived from different areas of active learning, that is, dynamic prediction, time series classification/analysis, and non-personalized recommendation. Also in the real-world simulation, we deal with heterogeneous types of drifts, that include abrupt, incremental, gradual, and reoccurring drifts, as well as some pseudo-stationary situations.

The rest of the paper is organized as follows—in Section 2, we illustrate the background of our work. We provide more details on the multi-armed bandit and concept drift problems, as well as a review of existing techniques in the context of non-stationary bandit. In Section 3, we give a formal description of the non-stationary multi-armed bandit problem and describe the methodological approach we propose to solve it. In Section 4 we illustrate the experimental setup and the different datasets considered for the evaluation and comparison purposes. Finally, in Section 5 we present and comment the results obtained by the different methods. Section 6 draws some conclusions and gives directions for future activity on this challenging research topic.

## 2. Background

In this section, we present the MAB problem and the main solution policies. Afterwards, we describe the problem of concept drift in active learning environments. Finally, a review of the state-of-the-art techniques related to MAB in non-stationary environment is provided.

### 2.1. Multi-Armed Bandit

Multi-Armed Bandit (MAB) is a powerful framework that allows agents to solve sequential decision making problems under uncertainty [16]. In the standard version, an algorithm has *K* possible actions (or arms) to choose from and *T* rounds (or time-steps). In each round, the algorithm chooses an arm, performs the associated action, and receives a reward for its choice. The reward is drawn independently from some fixed but unknown probability distribution, which depends only on the chosen arm. Thus, after each round the algorithm observes the reward for the chosen arm, but not for the other arms. The algorithm typically needs to explore: try out different arms to acquire new information in order to find the optimal arm in the given environment. Thus, a trade-off between exploration, that is, to explore different arms, and exploitation, that is, to exploit the acquired knowledge, is required in order to make optimal near-term decisions based on the available information. The goal of a MAB algorithm is to learn which arm is best (exploit its choices to maximize the collected reward), while not spending too much time exploring sub-optimal arms.

More formally, Multi-Armed Bandit (MAB) solves the exploration-exploitation dilemma in sequential decision problems [2]. Let K={1,⋯,K} be the set of arms that the agent can play and T={1,2,⋯,T} be the time instants; that is, at every time-step t∈T the agent has to select one of the *K* arms. Given a time-step t∈T and a chosen action k∈K (i.e., the agent selects arm *k*), the agent receives a reward $r_{k}\left(t\right)∼D_{k}$, drawn from an unknown probability distribution $D_{k}$. Given the reward $r_{k}\left(t\right)$ for arm *k*, the agent refines its estimation of the reward distribution $D_{k}$. In the specific case of a Bernoulli MAB, the reward is drawn from a Bernoulli distribution with mean $\mu_{k}=E\left[r_{k}\right]$. Thus, the outcome of every action is two-fold, failure or success, and the reward is $r_{k}\in \left\{0,1\right\}$. Given all the collected rewards up to time *t*, the agent updates its estimation $\tilde{\mu }{\left(t\right)}_{k}$ over the unknown expected value $\mu_{k}$ of the probability distribution associated with arm *k*. The best arm at any round *t* is the arm with highest expected reward, denoted as $\mu {\left(t\right)}^{*}=\max_{k\in K}\left\{\tilde{\mu }{\left(t\right)}_{k}\right\}$. Hence, the actual choice on which arm to choose next is made based on the estimated distribution and on the policy applied for arm selection. Different policies have been developed over the years to select the best arm to play at each round. These techniques differ in the way they handle the exploration-exploitation trade-off. The most studied in the scientific literature are:

- *ϵ-greedy*. Given a small value ϵ, the agent selects a random arm with probability ϵ and the best arm *a*, where $a\leftarrow argmax_{k\in K}\left\{\tilde{\mu }{\left(t\right)}_{k}\right\}$, with probability 1−ϵ.
- *Upper Confidence Bound*. Given a parameter α which controls the exploration-exploitation trade-off, the agent selects the best arm *a*, where $a\leftarrow argmax_{k\in K}\left\{\tilde{\mu }{\left(t\right)}_{k}+\alpha *S\left(\tilde{\sigma }{\left(t\right)}_{k}\right)\right\}$, and $S(\tilde{\sigma }{\left(t\right)}_{k})$ is proportional to the plug-in standard deviation for the reward distribution of arm *k* [17,18].
- *Thompson Sampling*. The agent selects the best arm *a*, where $a\leftarrow argmax_{k\in K}\left\{\hat{\mu }{\left(t\right)}_{k}\right\}$, and $\hat{\mu }{\left(t\right)}_{k}$ is a sample from the unknown reward distribution with the estimated expected value $\tilde{\mu }{\left(t\right)}_{k}$ [19,20,21].

For these approaches, many theoretic proofs of convergence to the optimal solution exist in the case of a stationary setting [22,23,24]. A stationary setting is defined as an environment in which the reward distribution $D_{k}$ for each arm *k* is assumed to be stationary (i.e., the distribution does not change) through all the time-steps in T.

In this work, we rely on the Bernoulli Thompson sampling version of the MAB formulation. This choice is motivated by a recent information-theoretic analysis of Thompson Sampling [25], which shows how regret bounds scale with the entropy of the optimal-action distribution. This analysis not only strengthens previous results and yields new insight into how information improves MAB performance, but it motivates the study of the interplay between MAB and entropy. A relevant contribution in this direction is offered by information-directed sampling [26], a new approach where each action is sampled to minimize the ratio between squared expected single-period regret and the mutual information between the optimal action and the next observation. It is worthwhile to mention that for the Bernoulli bandit problem, in the case of stationary environment, [26] demonstrates state-of-the-art simulation performance. Therefore, our choice to study, develop and apply the Bernoulli Thompson Sampling version of MAB, is further motivated by the fact that it can be modified to fully exploit the interplay between entropy and MAB when considering a non-stationary environment.

Through the years the MAB framework has been exploited by researchers to address different real-world applications. For instance, Scott [8] describes an heuristic for managing multi-armed bandits called randomized probability matching. It exploits this method to optimize for A/B testing in experimental design. A popular application of these techniques, from the early years, is the one on adaptive clinical trials [4], for which a review of the methodologies is illustrated in [27]. Another application of MAB and Gaussian optimization concerns the management of large network of sensors, such as the one presented by Srinivas et al. [28]. In the context of recommendation systems, many MAB applications have been presented, for the possibility to adapt them in online fashion. We mention the empirical evaluation on different bandit policies conducted in [9], and the hybrid UCB news recommender system based on Yahoo! data [10]. Finally, a peculiar application is presented in the work by Brochu et al. [29]. The authors apply MAB techniques to optimize the setting of the parameters in a procedural fluid animation system by showing the user examples of different parametrized animations and asking for feedback. This method exploits “prior” belief based on previous runs of the system and/or expert knowledge, to assist users in finding good parameter settings in as few steps as possible.

### 2.2. Concept Drift

In the context of online learning in dynamically changing environments, *concept drift* may happen whereas the distribution of the data stream shows a non-stationary behaviour. Concept drift refers to changes in the conditional distribution of the output (i.e., target variable) given the input (i.e., input features), while the distribution of the input may stay unchanged [13]. A typical example of concept drift is related to the change in user’s interests when following an online news stream. Whilst the distribution of the incoming news may remain the same, the conditional distribution of the interesting news for that user may evolve and change over time (e.g., following trending topics or periodic events). The idea of *active learning* is to adapt the learning model in order to cope with the changing distribution and promptly react to concept drift [13,30].

In a classification problem, given a data instance (X,y), we aim at predicting the target variable (class) $y\in \{y_{1},y_{2},\ldots ,y_{c}\}$ based on its *p*-dimensional set of features $X\in R^{p}$. Based on Bayesian Decision Theory, the classification problem is defined by the prior probabilities of the classes p(y) and the class conditional probability density functions (also known as likelihoods) p(X|y) for all classes $y=\{y_{1},y_{2},\ldots ,y_{c}\}$, where *c* is the number of classes. The classification decision is made according to the posterior probabilities of the classes, which for class $y_{i}$ is $p\left(y_{i}|X\right)\propto p\left(X,y_{i}\right)=p\left(X|y_{i}\right)p\left(y_{i}\right)$. Thus, given a stream of *T* classification instances $S=\{{\left(X,y\right)}_{t_{1}},{\left(X,y\right)}_{t_{2}},\ldots ,{\left(X,y\right)}_{t_{T}}\}$ concept drift between two time-points $t_{l}$ and $t_{m}$ formally occurs when:

(1) $$\exists \left(X,y\right):p_{t_{l}}\left(X,y\right)\neq p_{t_{m}}\left(X,y\right)$$

where $p_{t_{l}}\left(X,y\right)$ defines the joint distribution at time $t_{l}$ between the set of input variables X and the target class *y*. Thus, in a classification problem, the drift in Equation (1) may happen due to class priors p(y), or class conditional probabilities (likelihoods) p(X|y).

Please notice that, in this work, we are interested just in the first change, namely the one affecting the class prior p(y). This is due to the fact that in a standard multi-armed bandit the inference is based on the class (reward) distributions, while ignoring the possible input variables (i.e., covariate shift).

We further distinguish four different types of concept drift that may affect a stream of data [14].

- *Sudden/Abrupt*: the switch of concept p(y) happens abruptly, from time $t_{i}$ to the subsequent time $t_{i+1}$.
- *Incremental*: the switch of concept p(y) happens incrementally (slowly), with many smaller intermediate changes, from time $t_{i}$ to time $t_{i+n}$.
- *Gradual*: the switch of concept p(y) happens gradually, by switching back and forth to the old and new concept, from time $t_{i}$ to time $t_{i+n}$, before stabilizing to the new one.
- *Reocurring*: The switch of concept p(y) happens abruptly at time $t_{i}$, but after time $t_{i+n}$ the concept reverses back to the old one. It could be cyclic.

**Figure 1:**
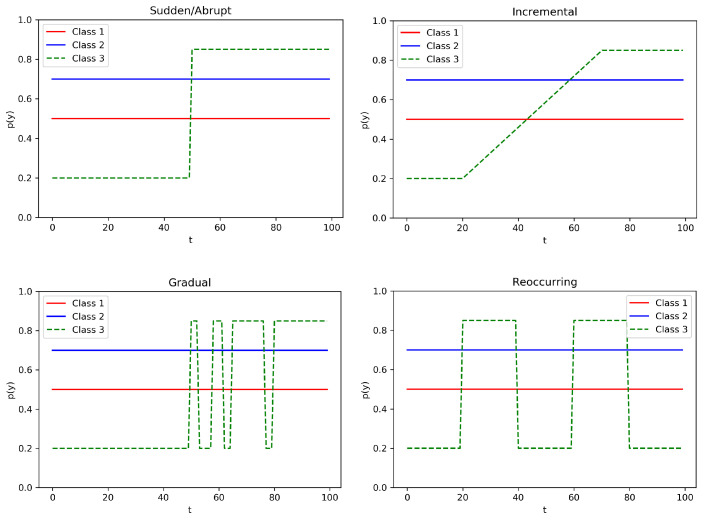
Examples of the four types of concept drift affecting the probability distribution of Class 3.

In Figure 1, we provide a representation of the four types of concept drift. In this illustrative example, we consider three classes, that is, *Class 1*, *Class 2*, and *Class 3*, with corresponding prior distributions p(y), evolving over time *t*: the distributions of *Class 1* and *Class 2* are not affected by any concept drift (i.e., they are stationary), while the prior distribution of *Class 3* presents in each graph a different type of concept drift (i.e., sudden/abrupt, incremental, gradual, and reoccurring). In our experimental analysis we consider all these different drift scenarios to provide a broader evaluation of the performances of the MAB algorithms.

Many algorithms and real-world applications were presented through the years, related to the problem of the concept drift. Most of them are summarized in literature reviews, such as [30,31]. For instance, [32] propose a set of statistical hypothesis methods in order to detect concept drift. In particular, the authors propose ad-hoc test statistics to be dynamically adapted to the multivariate data distribution—that is, a rank statistic on density estimates, and linear error margins induced by two classes of support vector machine (SVM). Classification through SVM is also proposed by [33] in order to handle the concept drift in a information filtering of textual data scenario. The key idea of this work is to automatically adjust the sliding window size, such that the estimated validation error is minimized. Again, a drift detection method that uses a statistical test of equal proportions (STEPD) to detect various types of concept drift is presented in [34]. A method based on an ensemble of classifiers for incremental learning of concept drift is instead described in [35]. The peculiarity of the proposed approach is that streaming data are processed through batches and a new classifier for each batch of data is trained. Then, these classifiers are combined using a dynamically weighted majority voting, based on time-adjusted accuracy on current and past batches.

### 2.3. Non-Stationary Multi-Armed Bandit

In this section we review the most relevant algorithms developed to solve the Non-Stationary MAB problem (i.e., under concept drift) which have been proposed and discussed in the specialized literature.

The two approaches inspiring the *f-dsw TS* algorithm are presented by Raj et al. [36] and Trovo et al. [15]. In the first one, the authors propose a *Discounted Thompson Sampling (dTS)* [36], which adds a discount to systematically increase the variance of the prior distribution and maintain exploration during time, reducing the effect of past observations. Instead, *Sliding Window Thompson Sampling (SW-TS)* [15] uses a sliding window approach to tackle, in a unified fashion, two different forms of non-stationarity: abruptly changing and incremental changing. This method exploits a sliding window of size *n* in order to process only the most recent *n* actions performed by the agent for the reward estimation. The paper provides the regret upper bounds on the dynamic pseudo-regret of SW-TS for the abruptly changing environment, for the smoothly changing one, and for the setting in which both of the above forms are present. Both algorithms adapt Thompson Sampling [19] to a Non-Stationary MAB. With respect to the UCB policy, Garivier et al. [37] introduce two kinds of UCB-like algorithms: *Discounted UCB* and *Sliding-Window UCB (SW-UCB)*. The former uses a discount to keep the exploration of the environment by gradually vanishing the older experiences while the latter uses a sliding window over the last *n* actions to completely forget older actions. A proof over both an upper-bound and a lower-bound for the expected regret are established, showing that both algorithms match the lower bound up to a logarithmic factor. An interesting approach, that extends the sliding window technique, is used in [38] to handle *Scaling MAB*. The goal addressed in the paper is to maximize cumulative rewards while minimizing the cost of selecting multiple arms per round. In this work, the authors suggest to combine Thompson Sampling with ADWIN [39], an adaptive sliding window method for change-point detection. This algorithm automatically grows the window size when it detects no changes and shrinks it when observing a change in the data.

Another class of algorithms uses a change point detection method to restart the agent’s knowledge when detecting a change in the environment. A good example is *Adapt-EvE*, proposed in [40], that uses an adaptive change point detection test based on Page-Hinkley statistics to detect abrupt changes in the environment. Other algorithms combine the use of different techniques. For example *Bayes-UCB* [41] combines a Bayesian approach with an UCB algorithm to unify different variants of UCB. This framework provides a unified view for several variants of the UCB algorithm, addressing different bandit problems, for example, parametric multi-armed bandits, Gaussian bandits with unknown mean and variance, linear bandits. Similarly, *Optimistic Bayesian Sampling (OBS)* [42] merges Thompson Sampling with a Bayesian approach. The probability of playing an action increases with the uncertainty in the estimation of the action value. Thus, it results in better directed exploratory behaviour. In a similar way many algorithms combine Change Point Detection with TS, for example, *Change-Point Thompson Sampling (CTS)* proposed in [43] uses a Bayesian Change Point Detection, and UCB like *Change Detection UCB (CD-UCB)* [44] and *GLR-klUCB* [45] uses a Bernoulli Generalized Likelihood Ratio Test in order to detect a change in the environment.

In the more specific context of *Referral Learning*,^46^] addresses the problem of time-evolving expertise in referral networks. Specifically an algorithm, called *Hybrid*, that combines *DIEL reset* with Thompson Sampling, or TS variants, is used in order to learn an effective referral strategy. For every action choice, Hybrid randomizes between one of the two selection strategies. In this case, non-stationary behaviour is handled by resetting the DIEL reset algorithm after each referral windows, while Thompson Sampling keeps trace of the entire experience gathered.

Finally, we mention different kinds of methods like *Differential Evolution (DE)*, proposed in [47], that uses an evolutionary approach. In this work the variation of the distributions depends on the number of times an option is evaluated rather than on the evolving time. This definition allows to apply these algorithms over a wide range of problems such as black-box portfolio selection. In [48] the authors introduce *SER4*, an algorithm that resets the reward estimators randomly during the game and then starts a new phase of optimization.

## 3. Methodology

In this section we outline the research methodology, formally define the problem and describe the proposed solving approach.

### 3.1. Problem Definition

We aim at solving the multi-armed bandit problem in a non-stationary setting (i.e., in presence of concept drifts). In the non-stationary setting we assume that the reward of the arms can change at every step, that is, $\mu_{t}^{k}$ no longer represents the whole sequence of rewards for one arm. We denote with $\mu^{k}$ the sequence of expected rewards for arm *k*: $\mu^{k}={\left\{\mu_{t}^{k}\right\}}_{t=1}^{T}$. Furthermore, we represent with P the family of admissible policies and let π∈P denote the candidate policy that, at every step, selects the arm to pull. At each time-step, the agent following the policy π selects an arm $I_{t}^{\pi }$ based on the initial prior *U* and past observations ${\left\{X_{n}^{I_{n}^{\pi }}\right\}}_{n=1}^{t-1}$. We denote the policy at the step *t* with $\pi_{t}$ as:

(2) $$\pi_{t}=\left\{\begin{array}{cc} \pi_{1}\left(U\right), & t=1\\ \pi_{t}\left(U,r_{1},r_{2},\cdots ,r_{t-1}\right), & t\geq 2. \end{array}\right.$$

In our formulation we focus on the Bernoulli TS approach. Specifically, we assume rewards to be drawn from a Bernoulli distribution. Thus, the TS algorithm represents the history of rewards as a Beta distribution for every arm, since it is the conjugate prior of the Bernoulli distribution. This distribution has two parameters: α and β. We update the Beta distribution of arm *k*, denoted with $B(\alpha_{k},\beta_{k})$, following the equation:

(3) $$B\left(\alpha_{k},\beta_{k}\right)=\left\{\begin{array}{cc} B(\alpha_{k},\beta_{k}), & \mathrm{if}\;I_{t}^{\pi }\neq k\\ B(\alpha_{k}+r_{t},\beta_{k}+1-r_{t}), & \mathrm{if}\;I_{t}^{\pi }=k, \end{array}\right.$$

where $I_{t}^{\pi }$ is the action selected at step *t* and, as said, $r_{t}$ is the reward at step *t*.

Furthermore, we need to define a metric to compare different policies in non-stationary environments. We decide to use a *dynamic oracle* [12] as the optimal policy to evaluate and compare *f-dsw TS* to state-of-the-art algorithms. The *dynamic oracle* optimizes the expected reward at each step t∈T by always selecting the best arm, with expected reward of $\mu_{t}^{*}$. Thus, we define the cumulative expected reward D(T) for the dynamic oracle at the last step *T*, as:

(4) $$D\left(T\right)=\sum_{t=1}^{T}\mu_{t}^{*}.$$

We define the *Regret* $R^{\pi }\left(T\right)$ for policy π at step *T*, as the difference between D(T) and the cumulative expected reward at step *T* for policy π:

(5) $$R^{\pi }\left(T\right)=\sum_{t=1}^{T}\mu_{t}^{*}-E_{\pi }^{\mu }\left[\sum_{t=1}^{T}r_{t}\right]=D\left(T\right)-E_{\pi }^{\mu }\left[\sum_{t=1}^{T}r_{t}\right],$$

where $E_{\pi }^{\mu }\left[\cdot \right]$ is taken over both the randomization of the policy and the randomization of the reward given by the environment.

### 3.2. f-Discounted-Sliding-Window Thompson Sampling

The MAB algorithm we propose, named *f-Discounted-Sliding-Window Thompson Sampling (f-dsw TS)*, mixes two different approaches: a discount approach, inspired by the work of [36,37] and a sliding window approach, as in [15,37]. Our solution implicitly assigns more relevance to the recent evidences from the environment, as in the sliding window approach, given that these rewards are not discounted. At the same time, the algorithm does not discard the older interactions by the agent, but it processes this history of rewards using a discount factor, which decreases the importance of older ones. Furthermore, the proposed sliding window method differs from the ones already presented in the literature given that each arm (i.e., each class of actions) independently processes its own sliding window. This optimization allows the agent to adapt to the specific drift happening on a single arm distribution, while maintaining the original information on the others. Thus, the *f-dsw TS* algorithm combines these two estimations of the reward: the estimation of the entire (discounted) history of the rewards and the most recent *n* rewards. We propose to aggregate the two views using a function f(.) that selects one of the two values (e.g., *max*, *min*) or mix them (e.g., *mean*). These approaches differ in how much the method is optimistic or pessimistic in the evaluation of different types of concept drift, as well as how aggressively it trade-offs exploration and exploitation in stationary situations. As an intuition, the *max* version is more sensitive to increasing drift, while being more conservative to decreasing drift in the expected reward function. This is due to the fact that the *max* function favours the higher estimation, which, in a increasing drift scenario, is provided by the sliding window method on the recent reward history. At the same time, in a situation where the expected reward function shows a decreasing drift, the *max* function follows the inertial optimistic estimation (i.e., the historic discounted trace), discarding pessimistic information about the decreasing reward, provided by the sliding window method. Opposite considerations hold for the *min* function. In Section 5.2, we provide further insights on the behavior of the algorithm variants, empirically derived from simulation in custom (controlled) environments. A complete and formal mathematical proof on the behavior (i.e., regret bounds) of the algorithm variants is outside the scope of this paper and it is left as future work.

More formally, we consider the Bernoulli MAB and we save the rewards with *K* Beta distributions (i.e., Bernoulli conjugate prior) where K=|K| is the number of arms in the environment. We call these Beta distributions *historic trace* denoted as $B(\alpha_{k},\beta_{k})$ because we save all the rewards received during the *T* steps. Furthermore, let γ∈(0,1] be the discount factor used to fade out the past observations in the historic trace. In addition, we define the *hot trace* denoted as $\overset{ˇ}{B}\left(\alpha_{k}^{n},\beta_{k}^{n}\right)$ where we maintain *K* Beta distributions based on the last *n* rewards taken from the arm *k*, where n∈[1,T] is the sliding-window size. We emphasize that, for every arm *k*, for the first r<n rewards received from it, the hot trace $\overset{ˇ}{B}\left(\alpha_{k}^{n},\beta_{k}^{n}\right)$ is formed with n=r.

The mix between the two components is performed before the selection of the arm to be played at step *t*. For every arm *k*, the algorithm computes an aggregated score $S_{k}\left(t\right)$, as:

(6) $$S_{k}\left(t\right)=f\left(\theta_{k}\left(t\right),{\overset{ˇ}{\theta }}_{k}\left(t\right)\right),$$

where f(.) is the aggregation function defined for the algorithm, $\theta_{k}\left(t\right)$ is a sample from the *historic trace* distribution $B(\alpha_{k},\beta_{k})$, ${\overset{ˇ}{\theta }}_{k}\left(t\right)$ is a sample from the *hot trace* distribution $\overset{ˇ}{B}\left(\alpha_{k}^{n},\beta_{k}^{n}\right)$ at step *t* for arm *k*. Finally, *f-dsw TS* chooses which arm to play at step *t*, as the one maximizing the aggregated score:

(7) $$I\left(t\right)=\underset{k}{arg max}\left(f\left(\theta_{k}\left(t\right),{\overset{ˇ}{\theta }}_{k}\left(t\right)\right)\right)=\underset{k}{arg max}\left(S_{k}\left(t\right)\right).$$

The complete pseudo-code of *f-dsw TS* is depicted in Algorithm 1. In lines (2–5), for each arm we sample a reward estimate from both *historic* and *hot* traces Beta distributions. In line (6) we apply the function f(.) to select one of the two estimates (or a mix of them) and choose the arm with the highest aggregated score. We apply the selected action (arm) and observe the reward at time *t* (i.e., $r_{t}$) in line (7). In line (8) we update the *historic trace* using the discount factor γ and in line (9) we update the *hot trace* in the same way as in the original Thompson Sampling, but using only the last *n* rewards collected by each arm.
**Algorithm 1:***f*-*Discounted-Sliding-Window TS*
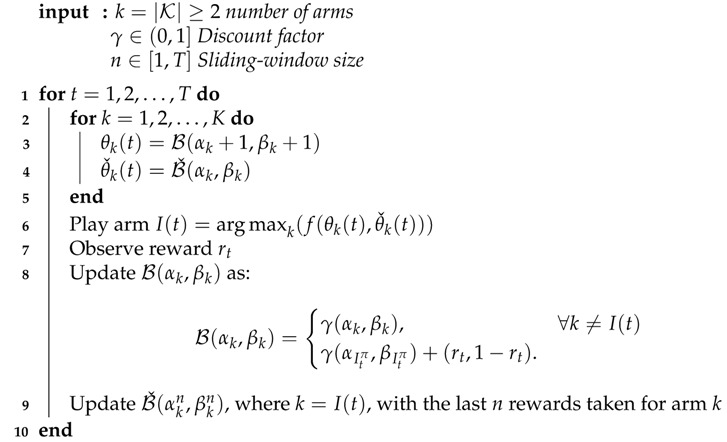


## 4. Experiments

In this section we report the structure of the experiments for evaluating the *f-dsw TS* algorithm in comparison with state-of-the-art algorithms (The full code of the experiments is available at https://github.com/CavenaghiEmanuele/Multi-armed-bandit). We first highlight the setup of our experimental campaign and then describe the characteristics of the data used in the evaluation (both synthetic and real-world). Finally, we illustrate the parameters tuning phase, that is, the choice of best parameters used in each experiment.

### 4.1. Experimental Setup

We test the *f-dsw TS* algorithm in different non stationary settings designed to evaluate different aspects. In particular, we consider both synthetic and real-world data. The two modalities of analysis slightly differ in how experiments are carried on. In the synthetic version, the environment is given (both in a custom or random way) and the reward distributions for each arm are defined (but unknown to the MAB algorithms). At time-step *t*, the algorithm selects an arm $a_{t}$ to pull and receive a stochastic reward $r_{t}$ sampled from the reward distribution for arm $a_{t}$, defined according to the environment. In this case, we can measure the performance of a MAB algorithm based on the theoretic dynamic regret (or just regret), as defined in Section 3. Another way to measure the actual performance of an algorithm *M* relies on the computation of the *cumulative reward* collected, defined as $CR\left(M\right)=\sum_{t=1}^{T}r_{t}$, or the *cumulative reward relative to the oracleO*, $RCR\left(M\right)=\frac{CR(M)}{CR(O)}$. Vice versa, in most real-world settings the theoretic reward distributions are hidden in the data, hence not accessible to the oracle. In this case, the evaluation of MAB algorithms is performed as follows—given an instance such as a user purchase of an online product in a real-world dataset, with a user feedback towards one action $f_{t}$, the algorithm selects which arm $a_{t}$ to pull (e.g., which product to recommend). If the algorithm selects the action for which the user provided positive feedback ($a_{t}==f_{t}$), then a positive reward $r_{t}=1$ is assigned to the algorithm, otherwise it receives a null reward $r_{t}=0$. Thus, the regret computation is not feasible and we must rely on a classification-based metric, that is, the accuracy at the end of the last round *T*. More formally for an algorithm *M*, $accuracy\left(M\right)=\frac{1}{T}\sum_{t=1}^{T}r_{t}$.

We compare different variants of the *f-dsw TS* algorithm with a set of state-of-the-art dynamic and stationary TS algorithms. In particular, in our experiments we investigate and compare the following algorithms:

- *Max-dsw TS*: *f-dsw TS* with *max* as an aggregation function *f*.
- *Min-dsw TS*: *f-dsw TS* with *min* as an aggregation function *f*.
- *Mean-dsw TS*: *f-dsw TS* with *mean* as an aggregation function *f*.
- *Discounted TS*: the TS enhanced with a discount factor, presented in [36]: the parameter γ controls the amount of discount.
- *Sliding Window TS*: the TS with a global sliding window, presented in [15]. The parameter *n* controls the size of the sliding window.
- *Thompson Sampling*: the standard Beta-Bernoulli TS.
- *Random*: a trivial baseline that selects each arm at random.
- *Oracle*: an oracle always selecting the best action at time *t*. It is exploited for regret computation.

### 4.2. Synthetic Datasets

In the synthetic evaluation, two different types of environments are designed—random-generated environments and custom environments.

#### 4.2.1. Random Environments

Firstly, we test *f-dsw TS* in randomly-generated environments, where the initial payoff probability for each arm is random and the drift probability is fixed. In this setting, each arm has a random initial reward distribution; the parameter of the Bernoulli reward distribution $\mu_{k}$ for each arm *k* is uniformly-sampled in [0,1]. Then, at each time-step *t*, each arm *k* has an independent drift probability d∈[0,1] of changing its reward distribution to a new Bernoulli distribution with uniformly-sampled parameter $\mu {\left(new\right)}_{k}\in \left[0,1\right]$. We set the number of classes (arms) to four and the number of time-steps to 1000, for every randomly-generated environment. For experimental purposes, we define a *random scenario* as a set of 1000 randomly-generated environments, with a fixed drift probability *d* and a fixed drift type (as defined in Section 2). The results for a random scenario are computed as the average results over each randomly-generated environment in that scenario. We propose a comparative evaluation of the MAB algorithms in different random scenarios. Specifically, we consider the following set of drift probabilities: d=[0.001,0.002,0.003,0.004,0.005,0.01,0.015,0.02] and the two main types of concept drift, namely abrupt and incremental.

Thus, we introduce two different types of random scenarios: *random-abrupt* and *random-incremental*. In the *random-abrupt* scenario, we compare the algorithms in different randomly-generated environments where the changes are abrupt; that is, at every time-step, for every arm *k*, the reward distribution with parameter $\mu_{k}$ has probability *d* to suddenly change to a new reward distribution with uniformly-sampled parameter $\mu {\left(new\right)}_{k}$, independently to the current reward distribution. In the *random-incremental* scenario, the algorithms are compared in different randomly-generated environments where the changes are incremental; that is, for every arm *k*, the reward distribution with parameter $\mu_{k}$ can start changing at every time-step with probability *d*. When arm *k* starts the drift, we uniformly sample a new reward distribution parameter, $\mu {\left(new\right)}_{k}$, and a rate of change, $\delta_{k}\in \left(0.001,0.01\right)$. Then, in the following time-steps, the arm *k* increments its distribution parameter by $\delta_{k}$ at each step, until it reaches $\mu {\left(new\right)}_{k}$. We do not allow further changes on arm *k* as long as a change is in progress on that arm.

#### 4.2.2. Custom Environments

**Figure 2:**
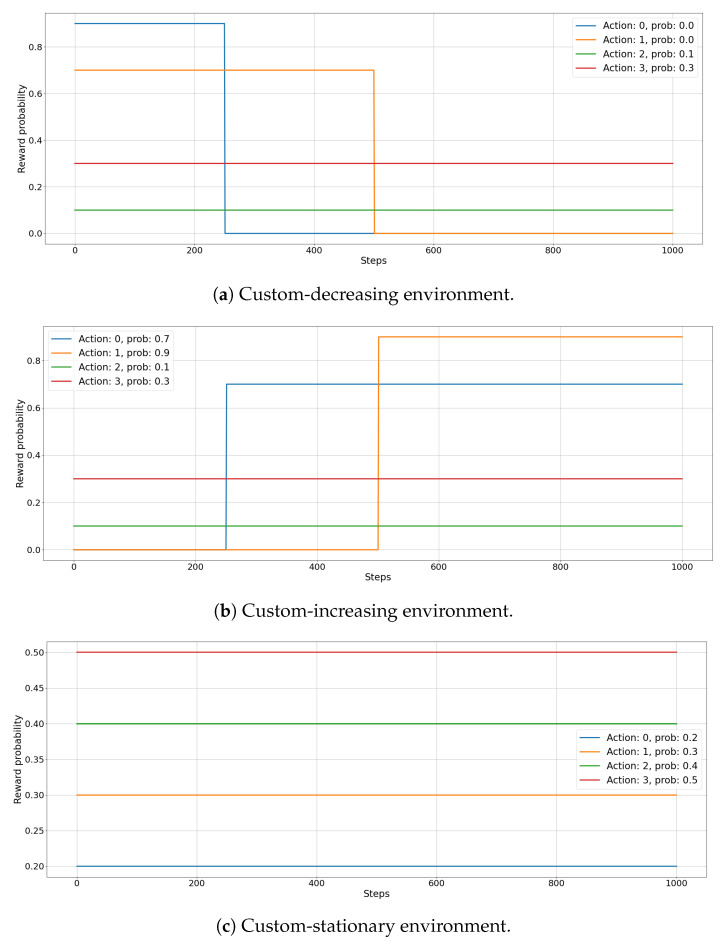
Reward distributions for *custom-decreasing* (**a**), *custom-increasing* (**b**), and *custom-stationary* (**c**) environments. Every function represents the evolution over time *t* of an arm reward probability in the custom environment.

In custom environments, the initial reward distribution and the changes are set by the experimenter. As before, the classes (arms) are four and each episode consists in 1000 steps. Results are averaged over 100 independent runs. The reward probability behaviour (i.e., the evolution of the Bernoulli parameter) of the three custom environments is depicted in Figure 2. This set of experiments is designed to investigate the behaviour of the *f-dsw TS* algorithm when the variability of real-world data is taken into account.

In the first scenario, called *custom-decreasing* Figure 2a, we test how the different MAB algorithms behave when the expected value of reward of the best arm suddenly decreases below that of competing arms, whose expected reward values stay stationary along time. In this situation, the agent has to discover the new best arm among those which did not changed their reward distributions. The initial reward distribution parameter μ for the four arms (Action 1, Action 2, Action 3, and Action 4) is set to 0.9,0.7,0.1,0.3 respectively. After 250 steps the expected reward for Action 1 drops to 0.0 and after 500 steps the expected reward for Action 2 becomes 0.0.

In the second scenario, called *custom-increasing* Figure 2b, we analyze the case of a data streaming where a sub-optimal action suddenly increases its expected value of reward, becoming the best solution, while others stay unchanged. Thus, the agent has to explore other arms to find the new optimal solution. Here, the initial reward distribution parameter μ for the four arms (Action 1, Action 2, Action 3, and Action 4) is set to 0.0,0.0,0.1,0.3 respectively. After 250 steps the expected reward for Action 1 increases to 0.7 and after 500 steps the expected reward for Action 2 becomes 0.9.

In the last scenario, called *custom-stationary* Figure 2c, we analyze the stationary case, where the reward distribution of every arm maintains its original value. In this way, we want to test how our solution performs when the environment is stable for a large amount of time between two changes in the reward distributions. In this case, the reward distribution parameter μ for the four arms (Action 1, Action 2, Action 3, and Action 4) are stationary to the values of 0.2,0.3,0.4,0.5 respectively, for all the 1000 steps.

### 4.3. Real Datasets

The second set of numerical experiments is devoted to test non-stationary MAB approaches on real-world data. Specifically, we adapted four online machine learning tasks to our framework. Namely, a prediction task on crimes in the city of Baltimore (*Baltimore Crime*), a classification task on insects species (*Insects* datasets collection), a recommendation task on local web-news (*Local News*), and a time-series analysis on microbial organisms in the tropical air ecosystem (*Air Microbes*). This collection of datasets presents heterogeneous characteristics in terms of number of instances, classes and time-granularity. Please, notice that we consider every instance in the dataset as a time-step of the MAB problem. The information related to each dataset is summarized in Table 1.

#### 4.3.1. Baltimore Crime

**Figure 3:**
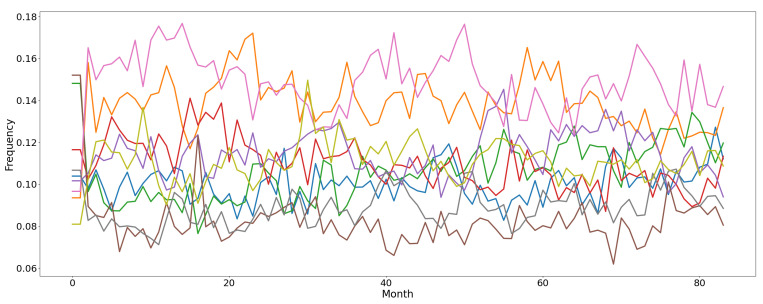
Monthly frequency of crimes in each district for the Baltimore Crime dataset. Every line represents the evolution of the relative frequency of crimes in each district, through the months.

The *Baltimore Crime* (https://data.baltimorecity.gov/search?q=crime%20data, accessed on 22 March 2021) dataset is about the crimes committed in the city of Baltimore (US), between 2014 and 2020. The dataset includes various features of the reported crime, such as the weapon used, the type of crime and its location. The monthly distribution of the crimes in the 9 city districts is depicted in Figure 3. Each function represents the crimes frequency in a specific city district, through the months. It may be noticed that some variability exists in the data, which exhibits few (gradual) concept drifts. Nevertheless, the fluctuations in the data streaming are much closer to some random noise than to actual concept drifts, as they are defined in the literature and represented in the artificially-generated environments.

The task we want to accomplish with *f-dsw TS* is to predict where the next crime is happening (i.e., in which district of the city), based on the past history. This prediction is relevant to help the local police department in organizing the allocation of the patrols. Thus, we map each of the 9 districts of the city to an arm, in order to predict where a crime will be committed at every step. If the predicted district at time-step *t* corresponds to the one in which the crime actually happened, a positive reward of 1 is provided, 0 otherwise.

#### 4.3.2. Insects

The *Insects* collection, introduced in [49], is composed by 11 independent open-source datasets with different properties, to evaluate stream classifiers and drift detectors (https://sites.google.com/view/uspdsrepository, accessed on 22 March 2021). The datasets are based on a real-world streaming application, which takes advantage of the use of optical sensors to recognize flying insect species in real-time. To build the insect stream datasets with concept drifts, the data from different species were collected by using optical sensor in a non-stationary controlled environment for three months approximately. The drifts on the insect species distribution were physically induced by modifying temperature conditions within the environment. They collected around one million instances for 17 different insect species. Afterwards, they post-processed the data by reducing the number of classes to the most frequent 6 (three species of both sexes) and they provided two versions of each dataset type—one with the original (imbalanced) class distribution and one with an artificially balanced class distribution. In our experiments we exploit the imbalanced versions of four of these datasets, namely the ones with *incremental*, *abrupt*, *incremental-abrupt-reoccurring*, and *incremental-reoccurring* concept drifts. For a detailed description of each dataset, please refer to the original paper [49]. Thus, the task here is to predict at each point in time which class of insect has been detected by the sensors. Each arm is mapped to one of the 6 insects classes; if the selected arm corresponds to the actual insect class recorded, a positive reward of 1 is provided, 0 otherwise.

#### 4.3.3. Local News

**Figure 4:**
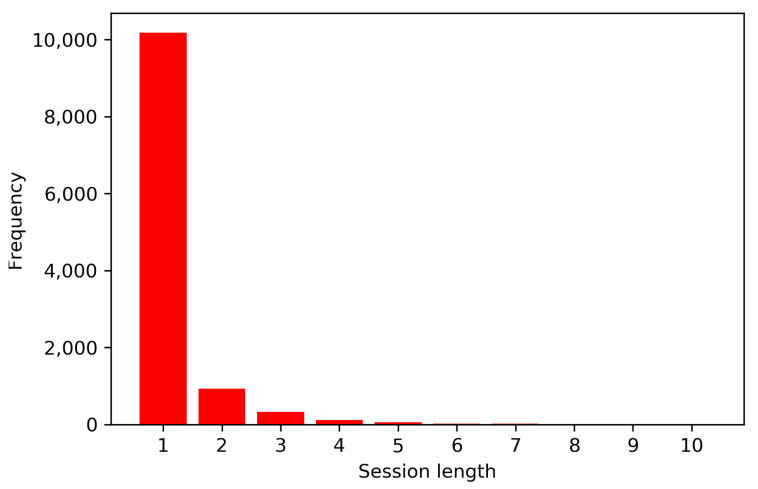
Histogram of the session length in the Local News dataset.

The *Local News* dataset is a private dataset, presented for the first time in [50]. The dataset includes the logged interactions to an Italian local news website, for an entire year (from 1 April 2016 to 30 March 2017). All the accesses to the news website are in anonymous form, thus, only interactions within the same browsing session could be personalized. Nevertheless, the length of these sessions is usually very short, with more than 85% of sessions represented by just one interaction, with an average session length of 1.23 interactions. The distribution of session length is represented in the graph of Figure 4.

**Figure 5:**
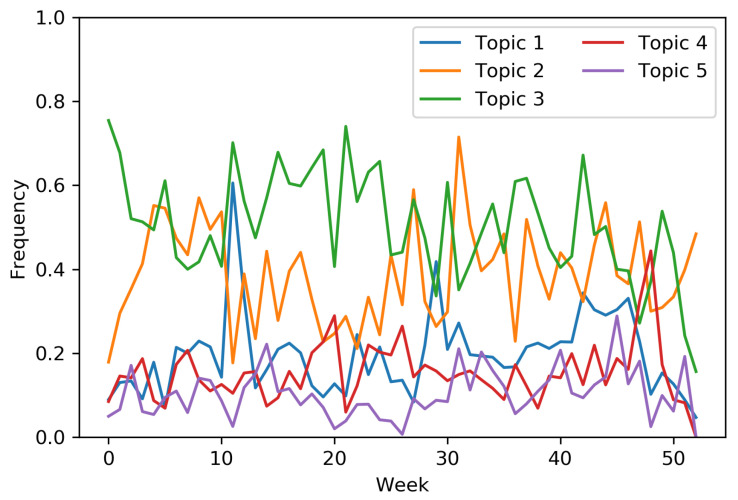
Frequency of the weekly topic interactions for the Local News dataset.

Together with each news article a snippet of the news content was provided. Therefore, we were able to apply a *Latent Dirichlet Allocation (LDA)* topic model [51], to extract 5 topics from the article corpus. Hence, each article was represented by a probabilistic distribution over the topics. We binarize the topic representation of each news article by retaining those topics with a relevance of above 10% for that article. Hence, an article could be mapped to more than one topic. The relative frequency of the topic interactions (and thus the drifting concepts) through the weeks is represented in Figure 5. From the graph, it could be easily noticed how concept drifts affect this data stream, that is, the sudden drift of *Topic 1* after the 10th week and the gradual drift of *Topic 2* distribution between the 20th and the 30th week. Nevertheless, some other topic distributions show an evolution that is more similar to a stationary behavior with some noise variance (i.e., *Topic 3* and *Topic 4*). We apply the MAB approach to construct a non-personalized recommendation system for the news topics. Namely, at each time step (i.e., user interaction) we select a topic to recommend to the user; each topic is mapped to an arm choose by the MAB algorithm. If the selected arm corresponds to one of the topics associated to the article the user interacted with, a positive reward of 1 is provided, 0 otherwise.

#### 4.3.4. Air Microbes

**Figure 6:**
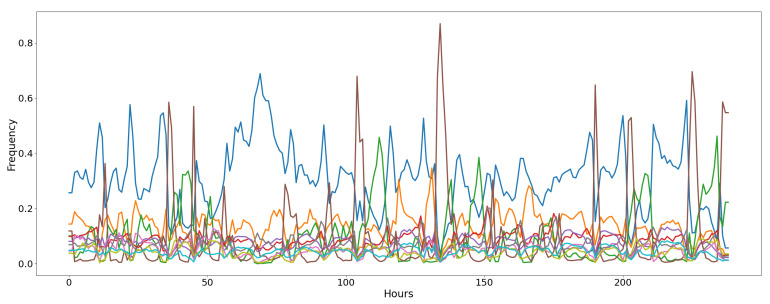
Frequency of the classes of air microbial organisms for the Air Microbes dataset. Every line represents the evolution of the relative frequency of a microbe class every two hours for 20 days.

The *Air Microbes* dataset, presented in [52], includes observations of microbial communities in the tropical air ecosystem. The data are collected along five weeks (each composed by five days); we consider only the last four weeks, given that the first one was used for instruments calibration and it is subjected to measurement errors. Every sample was gathered every two hours from three different sequencing devices positioned on an airplane. Hence, every observation in the raw dataset is represented by three counts (one for each device) of the number of occurrences for each microbial community. The number of communities (or classes) considered in the dataset is 10. We pre-processed the dataset to adapt it to the MAB evaluation. Firstly, we average the three frequency counts to obtain a single time-series for each community. Then, we convert the absolute count into a relative frequency of each community. The graph representing the evolving relative frequency of each microbial community is depicted in Figure 6. From this representation it can be noticed how concept drifts naturally occur in the data distribution. For example, several abrupt, incremental, and reoccurring drifts can be spotted due to changing atmospheric conditions (i.e., day and night alternation or air humidity variation). We exploit this computed relative frequency for each class as an estimation of the theoretic expected reward (i.e., the *p* parameter of a Bernoulli distribution), and we map each class into a MAB arm. At each time-step (i.e., one minute), we select an arm (i.e., a predicted microbes class) and we sample from the current estimated reward distribution for that arm a stochastic reward (1 for success, or 0 for failure). Please notice that we keep fixed the two-hours frequency value for 120 steps in order to simulate a sample every minute.

### 4.4. Parameters Tuning

The MAB algorithms depend of two parameters, namely γ, that is, the discount factor for the reward estimation, and *n*, that is, the sliding window size. Therefore, to find their optimal values in different environments we performed parameters tuning.

For *random-abrupt* and *random-incremental* settings, we search the optimal parameters when setting p=0.005, on 100 environments with 20 test each. The results are shown in Table 2.

For real-world datasets, we retain a separate portion of the dataset (validation set) and run the evaluation to select the best set of parameters for that task. For the Baltimore Crime and Local News dataset, the validation set consists in the first 20% of the original dataset, for the Insects datasets we optimize the parameters over an independent dataset of the Insects collection, called *incremental-gradual*. Finally, for the Bacteria dataset we use the second week to select the best parameters. The results of the tuning for real-world datasets are shown in Table 3.

## 5. Results

### 5.1. Random Environments

We report the results of the MAB algorithms on random scenarios with abrupt and incremental concept drift, as described in Section 4. We test how MAB algorithms behave when different rates of change *d* are considered. The performance of MAB algorithms are measured with the cumulative reward relative to the oracle, defined as $RCR\left(M\right)=\frac{CR(M)}{CR(O)}$ where CR(M) is the cumulative reward of algorithm *M* as described in Section 4.1.

**Figure 7:**
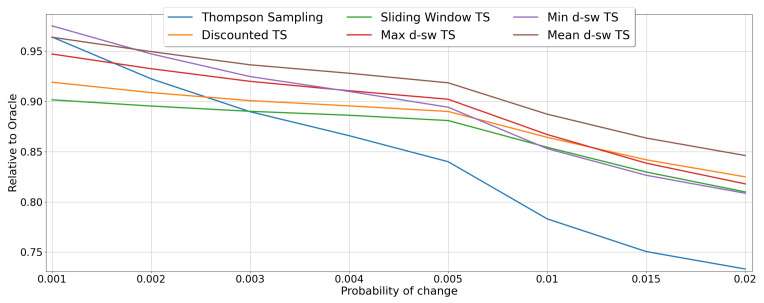
Results of the MAB algorithms in environments with abrupt changes. Line plots represent the performance of each algorithm in terms of *RCR*, when the probability of drift *d* varies.

The results for the *random-abrupt* scenario under different values of the drift probability *d* are reported in the Figure 7. Generally, the best performing algorithm is the *mean-dsw TS*, which achieves an *RCR* of around 96% in the most stationary scenario with probability of change d=0.001, whereas it scores a relative cumulative reward of 85% in the situation where the changes occurs with d=0.02. A similar behavior is showed by the algorithm *max-dsw TS*, while the pessimistic version *min-dsw TS*, seems to be impacted the most by the increased probability of change, being the overall best for d=0.001, but performing worse than the two baselines *sliding window TS* and *discounted TS* for the case of d=0.02. As expected, when *d* increases the performance of the algorithms degenerates, with the original TS algorithm achieving the worst performance (from around 96% for d=0.001 to less than 74% for d=0.02).

**Figure 8:**
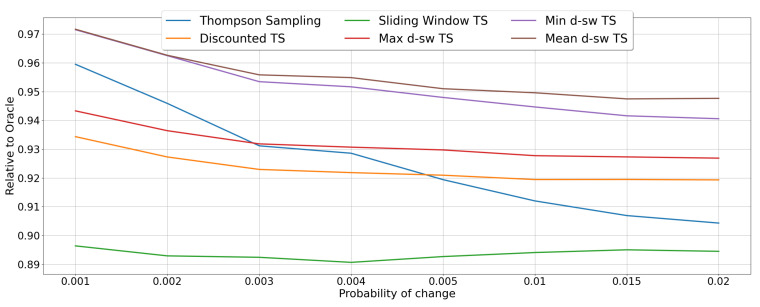
Results of the MAB algorithms in random environments with incremental changes. Line plots represent the performance of each algorithm in terms of *RCR*, when the drift probability *d* varies.

In Figure 8 we show the results for MAB algorithms in the *random-incremental* scenarios under different values of drift probability *d*. Again, the best performances are obtained by *mean-dsw TS* that achieves the maximum for lower values of *d* (*RCR* of 97% when d=0.001), while it stays constantly above 94% for all the considered values of *d*. In this setup, *min-dsw TS* scores very closely to the best performing one in all the considered scenarios. Again, *Thompson Sampling* shows a drastic drop when *d* is increasing, going from a 96%, with d=0.001, to below 91%, with d=0.02. Nevertheless, it is the *sliding window* baseline which achieves the worst performance, being constantly below 90% of relative cumulative reward. This finding proves how the sliding window method alone is not optimal in situation where incremental concept drift occurs. Finally, *max-dsw TS* and the other baseline *discounted TS* perform very similarly, being outperformed by standard TS for similar values of *d*, but achieving better results for greater values of *d* (i.e., showing more robustness when the changes are more frequent).

### 5.2. Custom Environments

We provide the results, achieved by the tested algorithms, in the three custom environments described in Section 4. In this case we provide two metrics, namely the regret (as defined in Section 3) and the cumulative reward, achieved by each algorithm along the 1000 rounds of iteration. Please notice that the presented results are averaged over 100 replications.

**Figure 9:**
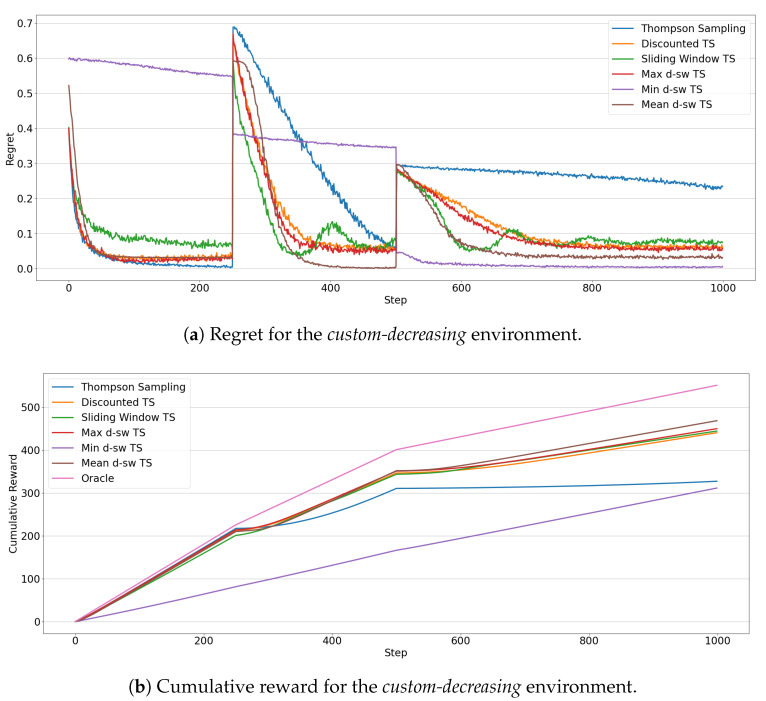
Results of the MAB algorithms in the *custom-decreasing* environment. Line plots represent the regret (**a**) and the cumulative reward (**b**) through 1000 steps for each algorithms.

Figure 9 illustrates the results for the *custom-decreasing* environment. The regret evolution of the algorithms (Figure 9a) highlights how non-stationary algorithms are faster to adapt to the first sudden drift. In particular, *mean-dsw TS* and *max-dsw TS* achieve the smallest regret at the end of the first phase. After the second drift, in the last 450 rounds Thompson Sampling completely fails to adapt to the change, showing a linearly decreasing regret, while *min-dsw TS*, *mean-dsw TS*, and *max-dsw TS* get faster to the smallest regret in the pool. Interesting to notice the peculiar behavior of *min-dsw TS* in the three phases of the environment. Before the second drift, the algorithm is the worst performing, showing a regret curve that linearly decreases without converging to the optimal results of the competitors. This testifies the fact that, in the initial stationary situation, the algorithm keeps exploring sub-optimal arms, failing to exploit the optimal arm choice. After the second drift, instead, *min-dsw TS* achieves the lowest regret, outperforming all other algorithms. An explanation on this behavior could be found in the different magnitude of the expected rewards of optimal and sub-optimal arms in the three phases of *custom-decreasing* environment (as depicted in Figure 2a). These comments reflect also in the measured cumulative reward for the competing MAB algorithms (Figure 9b). *Mean-dsw TS* shows the best overall cumulative reward after the last change, with the two non-stationary baselines (*Discounted TS* and *Sliding Window TS*) being close to *max-dsw TS*. The standard version of *Thompson Sampling* suffers a significant decrease of its performances after the last change, while *min-dsw TS* shows a linear increase of its cumulative reward, being unaffected by the changes in the environment.

**Figure 10:**
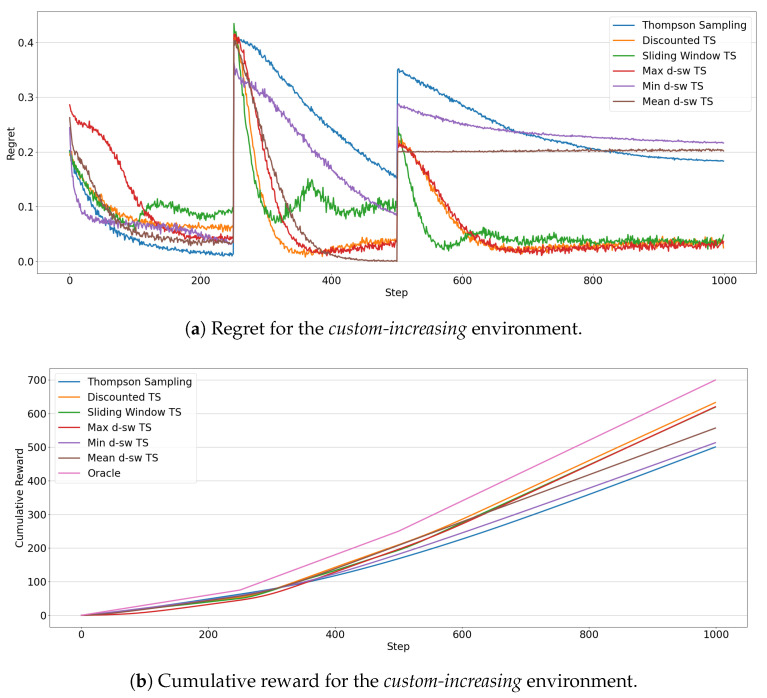
Results of the MAB algorithms in the *custom-increasing* environment. Line plots represent the regret (**a**) and the cumulative reward (**b**) through 1000 steps for each algorithms.

In the *custom-increasing* scenario the results, which are shown in Figure 10, witness how these are the most difficult drift types for the *f*-dsw TS algorithms. Figure 10b shows that *Discounted TS* achieves the best cumulative reward with *max-dsw TS* and *Sliding Window* being close to it. *Min-dsw TS* achieves a cumulative regret slightly greater than the standard version of *Thompson Sampling*, while *mean-dsw TS* performs better than these last two approaches, but worse than the best performing ones. The regret in Figure 10a proves that the *min-dsw TS* algorithm is able to adapt to the changes just slightly better than the standard version of *Thompson Sampling*, justifying the cumulative reward observed. The other algorithms adapt in a similar way, except for the *mean-dsw TS* in the second drift, which it does not detect at all the change in the rewards distributions. Similar observations to the *custom-decreasing* situation can be drawn for the *min-dsw TS*, but in reversed order. The algorithm starts to exploit the optimal arm in the first phase, while it explores sub-optimal arms after the second drift (i.e., after the increment of the expected reward of two arms). Please notice that, in both custom environments, *max-dsw TS* is always able to adapt to the drifts, regardless of their entity. As expected, in the *custom-increasing* situation it achieves the overall lowest regret, after the two drifts.

**Figure 11:**
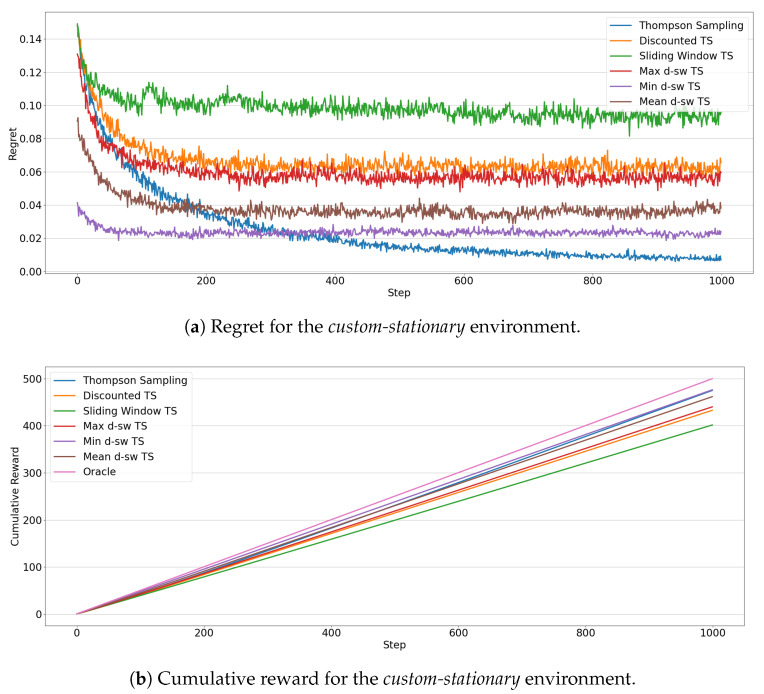
Results of the MAB algorithms in the *custom-stationary* environment. Line plots represent the regret (**a**) and the cumulative reward (**b**) through 1000 steps for each algorithms.

Finally, Figure 11 shows the results for the *custom-stationary* environments, that is, when the reward distributions do not change over the time, for example between two changes. The agents that achieve the best cumulative reward (Figure 11b), are *min-dsw TS* and, as expected, the original version of *Thompson Sampling*, due to the fact that this stationary algorithm does not spend time to explore the environment when it has already tried all arms enough times. The analysis of the regret in Figure 11a shows that *min-dsw TS* is the fastest to find the best arm and then it achieves a lower regret during time compared to all other non-stationary algorithms. Contrary to *custom-increasing* environment, *max-dsw TS* and *Discounted TS* perform worse than other algorithms, while *Sliding Window* is the worst in this scenario. The *mean-dsw TS* achieves, as expected, average results compared to the other competing algorithms.

### 5.3. Real-World Environments

In Table 4 we summarize the results on the different real-world datasets for each algorithm. In this case, given that a real-world task does not come together with true reward distributions for each action, we change the reference metric to the classification accuracy registered at the end of the last round—that is, the number of correctly guessed instances (or the cumulative reward), divided by the number of rounds *T*. The only exception is the *Air Microbes* dataset, for which accuracy computation is unfeasible, given that we do not have access to instance classes. In this case, we estimate a hourly reward distribution for each arm (based on hourly class frequency) in order to compute the relative cumulative reward at the *T*-th round, as for synthetic data. The results derive from the average over 10 replicates. In Appendix A we report the box plots of the collected results for each real-world task.

From Table 4, it emerges that the *min-dsw TS* version of *f-dsw TS* outperforms other algorithms on every real-world task.

Specifically, in the *Baltimore Crime* dataset *min-dsw TS* achieves an accuracy of 14.61% which is close to the 14.55% achieved by the standard TS baseline, and the 14.48% achieved by the *mean-dsw TS*. In this task the evolution of the concepts differs from the canonical definition of concept drift (i.e., the variability in the data streaming is similar to random noise), which makes it closer to a pseudo-stationary scenario. The algorithms with a more aggressive behavior towards drifts are negatively affected by this data structure and thus they achieve significantly worse performance values than those achieved by *min-dsw TS* (i.e., below 14.2% for *max-dsw TS*, sliding window, and discounted baselines).

For the collection of *Insects* datasets, similar conclusions can be drawn, with *min-dsw* and *mean-dsw* always outperforming the non-stationary baselines *D-TS* and *SW-TS* in terms of accuracy. *Max-dsw TS* is generally in line with the performances of the non-stationary competitors. In more details, the *insects-abrupt* dataset registers the smallest accuracy value for the standard TS algorithm, being greatly affected by the sudden drifts. At the same time, *mean-dsw TS* shines by achieving a good accuracy value (40.21%), very close to the dataset-best of 40.24%, achieved by *min-dsw TS*. Also *SW-TS* gets the best result of 39.94% among the baselines performances on the insects collection. As a general remark, all the non-stationary algorithms seem to perform better in scenarios with abrupt rather than incremental changes. The only exception is again the best performing method *min-dsw* which achieves slightly better accuracy on the *insects-incremental* (40.47%) and *insects-incremental-reoccurring* (40.3%).

The *local news* dataset appears to be the least difficult challenge, as testified by the highest accuracy achieved by the random baseline (23.69%). Furthermore, Thompson Sampling, with an accuracy of around 51.5% significantly outperforms *Sliding Window TS*, as well as *max-dsw TS*, and being close to the performance of *Discounted TS*. The reasons for this are similar to the ones mentioned for the *Baltimore Crime* dataset on the pseudo-stationary nature of data. Nevertheless, *min-dsw* is once more the best performing method with an accuracy of 53.7% (+2.2% w.r.t. TS), followed by *mean-dsw* (53.17%).

Finally, the findings are once more confirmed for the *Air Microbes* task. In fact, *min-dsw* significantly outperforms the other MAB algorithms, achieving a RCR above 86%, whereas the scores of the TS baselines and other *f*-dsw versions lie between 81% and 83% of RCR. *Mean-dsw* is confirmed to be the second best MAB algorithm in the pool, as the only other algorithm to perform with an RCR greater than 82%.

## 6. Conclusions

In this paper, we introduced and analyzed a new Multi-Armed Bandit (MAB) algorithm, called *f-Discounted-Sliding-Window Thompson Sampling* (*f-dsw TS*), for non-stationary environments, that is, when the data streaming is affected by concept drift. The *f-dsw TS* algorithm is based on Thompson Sampling (TS) and exploits a discount factor on the reward history and an arm-related sliding window to contrast concept drift in non-stationary environments. We investigated how to combine these two sources of information, namely the discount factor and the sliding window, by the mean of an aggregation function f(.). In particular, we proposed a pessimistic (f=min), an optimistic (f=max), as well as an averaged (f=mean) version of the *f-dsw TS* algorithm. A rich set of numerical experiments was performed to compare the performance of the *f-dsw TS* algorithm to both stationary and non-stationary state-of-the-art TS algorithms. We exploited synthetic environments (both randomly-generated and custom) to analyze and compare the performance of the *f-dsw TS* algorithm to state-of-the art baselines under different types of drift, i.e, sudden/abrupt, incremental, gradual, increasing/decreasing drift. Furthermore, we adapted four active learning tasks to our framework: a prediction task on crimes in the city of Baltimore (*Baltimore Crime*), a classification task on insects species (*Insects* datasets collection), a recommendation task on local web-news (*Local News*), and a time-series analysis on microbial organisms in the tropical air ecosystem (*Air Microbes*). The *f-dsw TS* approach emerged as the best performing MAB algorithm. At least one of the versions of *f-dsw TS* performed better than the baselines in synthetic environments, proving the robustness of *f-dsw TS* under different concept drift types. Moreover, the pessimistic version (f=min) resulted as the most effective in all real-world tasks.

Future directions of the presented work are manifold. We aim at extending the approach introduced for *f-dsw TS* to other MAB policies, namely ϵ-greedy and Upper Confidence Bound (UCB). Furthermore, we are going to analyze the theoretic bounds over the expected regret/reward of the *f-dsw TS* algorithm. Finally, we consider important to investigate the adoption of different reward distributions (i.e., Gaussian). This will allow to include contextual information (i.e., features/attributes) in our framework and hence to improve its effectiveness in real-world prediction and classification tasks.

## Figures and Tables

**Table 1:** Meta-information for each real-world dataset. We provide the number of classes (i.e., the number of actions/arms in the Multi-Armed Bandit (MAB) setting), the number of instances (i.e., the time-steps in the MAB setting), and the actual time span in which data were collected.

Dataset	Classes	Instances	Time Span
Baltimore Crime	9	321,147	6 years
Insects-Incremental	6	452,045	3 months
Insects-Abrupt	6	355,276	3 months
Insects-Incremental-gradual	6	143,224	3 months
Insects-Incremental-abrupt-reoccurring	6	452,045	3 months
Insects-Incremental-reoccurring	6	452,045	3 months
Local News	5	13,526	1 year
Air Microbes	10	28,560	20 days

**Table 2:** Best parameters selected after the tuning phase for *random-abrupt* and *random-incremental* scenarios.

Algorithm	Random-Abrupt	Random-Incremental
Max-dsw TS	γ=0.99 n=25	γ=0.99 n=50
Min-dsw TS	γ=0.95 n=100	γ=0.95 n=75
Mean-dsw TS	γ=0.95 n=25	γ=0.99 n=50
Discounted TS	γ=0.98	γ=0.99
Sliding Window TS	n=100	n=100

**Table 3:** Best parameters selected after the tuning phase for real-world datasets, that is, *Baltimore Crime*, *Insects*, *Local News* and *Air Microbes*.

Algorithm	Baltimore Crime	Insects	Local News	Air Microbes
Max-dsw TS	γ=0.9999 n=800	γ=0.999 n=800	γ=0.999 n=800	γ=0.9999 n=800
Min-dsw TS	γ=0.999 n=800	γ=0.99 n=200	γ=0.95 n=800	γ=0.99 n=800
Mean-dsw TS	γ=0.9999 n=800	γ=0.999 n=800	γ=0.99 n=400	γ=0.9999 n=800
D-TS	γ=0.9999	γ=0.999	γ=0.999	γ=0.9999
SW-TS	n=12,800	n=3200	n=3200	n=12,800

**Table 4:** Comparison of performances (%) of MAB algorithms for real-world datasets. * All tasks are evaluated in terms of classification accuracy, except for *Air Microbes* which is evaluated in terms of cumulative reward relative to the oracle (*RCR*). The best performance for each dataset is indicated in bold.

Dataset	Max-dsw	Min-dsw	Mean-dsw	D-TS	SW-TS	TS	Rand
Baltimore Crime	14.18	**14.61**	14.48	14.11	14.18	14.55	10.85
Insects abrupt	39.30	**40.24**	40.21	39.22	39.94	28.8	16.65
Insects incremental	38.94	**40.47**	39.94	38.82	39.72	35.35	16.63
Insects incr-abrupt-reoc	38.54	**40.10**	39.44	38.45	38.77	33.05	16.69
Insects incremental-reoc	38.58	**40.30**	39.53	38.49	38.63	33.48	16.68
Local News	50.94	**53.70**	53.17	51.46	50.79	51.49	23.69
Air Microbes *	81.13	**86.06**	82.57	80.93	81.42	81.94	26.95

**Figure A1:**
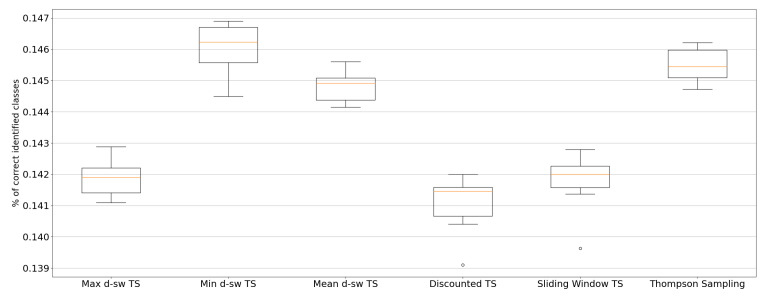
Accuracy results over 10 replications for the Baltimore Crime dataset.

**Figure A2:**
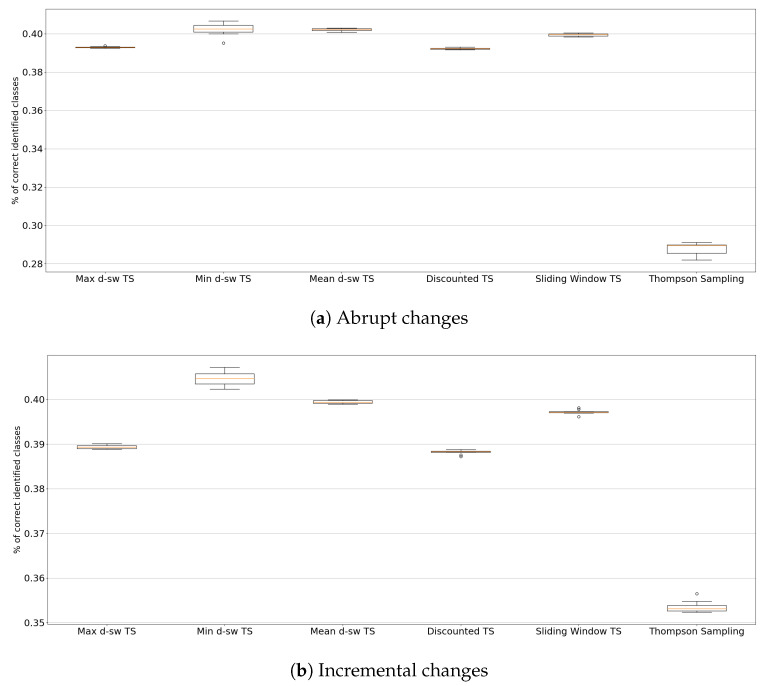

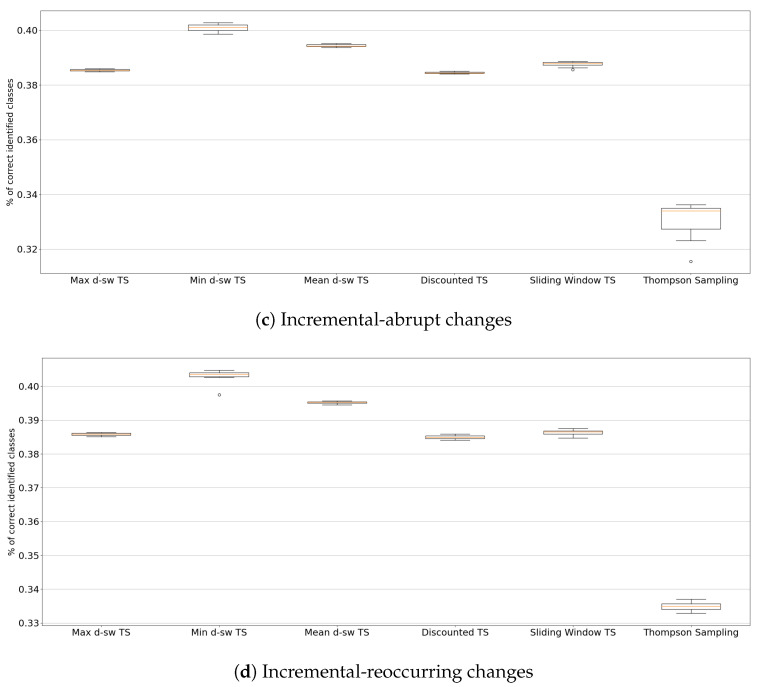
Accuracy results over 10 replications for the Insects datasets collection.

**Figure A3:**
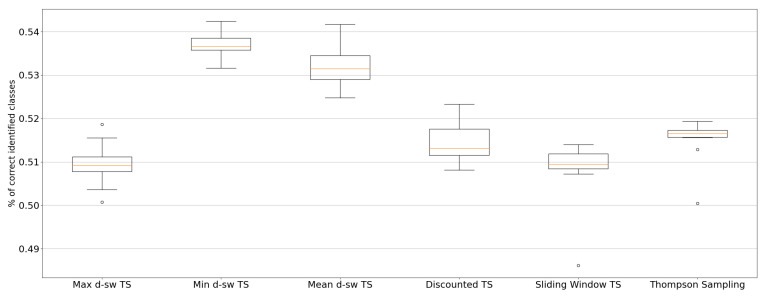
Accuracy results over 10 replications for the Local News dataset.

**Figure A4:**
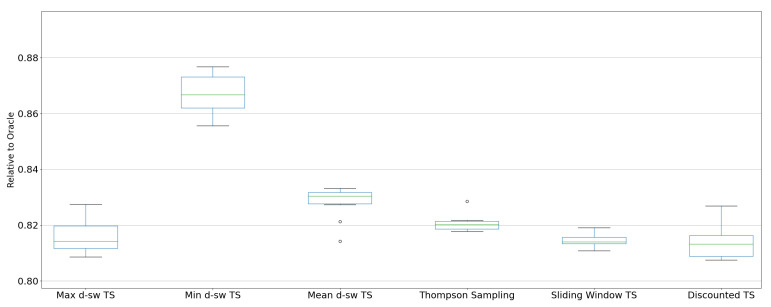
Relative cumulative reward (RCR) results over 10 replications for the Bacteria dataset.

## Data Availability

No new data were created or analyzed in this study. Data sharing is not applicable to this article.

## Appendix A

Here we provide the boxplots of the results for the experiments on real-world datasets, shown in Table 4.

In Figure A1 we show the accuracy results for the Baltimore Crime dataset.

In Figure A2 we present the accuracy of the tested algorithms for every drift in the Insect dataset: abrupt Figure A2a, incremental Figure A2b, incremental-abrupt Figure A2c and incremental-reoccurring Figure A2d.

In Figure A3 we report the boxplot of the accuracy for the Local News dataset.

Finally, in Figure A4, we show the results in terms of relative cumulative reward *(RCR)* for the Air Microbes dataset.
